# Cellular Senescence in the Regenerative Niche Hampers Skeletal Muscle Repair

**DOI:** 10.14336/AD.2024.1501

**Published:** 2025-02-21

**Authors:** MingYu Qiu, YangYang Li, QiSen Wang, XiaoTing Jian, JingWen Huang, WeiChao Gui, Jijie Hu, Hua Liao

**Affiliations:** ^1^Guangdong Provincial Key Laboratory of Construction and Detection in Tissue Engineering, Department of Anatomy, School of Basic Medical Science, Southern Medical University, Guangzhou, China.; ^2^Clinical Medicine in 8-Year Program, Southern Medical University, Guangzhou, China.; ^3^Department of Orthopaedics and Traumatology, Nanfang Hospital, Southern Medical University, Guangzhou, 510515, China.

**Keywords:** regeneration, skeletal muscle, senescence, cell niche, signaling pathway

## Abstract

With the growing interest in skeletal muscle diseases, understanding the processes, factors, and treatments associated with muscle regeneration is crucial. Skeletal muscle regeneration is a complex process that largely depends on the niche composed of cell populations, such as satellite cells, and their microenvironment. Cellular senescence is associated with various physiological processes and age-related diseases and plays a significant role in the muscle regeneration niche. Deciphering senescence-associated alterations within this niche provides critical insights for developing targeted anti-aging therapies. This review synthesizes recent studies to elucidate the composition of the niche and its cell-cell interactions and outlines the effects of aging on muscle regeneration and corresponding therapeutic strategies. This review summarizes emerging findings and technologies in muscle regeneration, analyzing therapeutic potential and limitations of current approaches for age-related conditions to support research advancement.

## 1. Introduction

Skeletal muscle regeneration is orchestrated by a specialized niche that coordinates cellular and molecular events during tissue repair. After muscle injury, niche signaling alterations activate satellite cells. Activated satellite cells cooperate with niche components to reconstruct muscle fibers. The muscle regenerative niche is a specialized microenvironment that facilitates muscle regeneration and repair, including diverse cell types, the extracellular matrix (ECM), blood vessels, neural networks, and cellular factors [1].

Cellular senescence is a fundamental biological process, significantly affecting the process of muscle regeneration. Senescent cells exhibit distinct hallmarks: irreversible cell cycle arrest, altered gene expression, activated tumor suppressor pathways, chromatin remodeling, apoptosis resistance, and elevated protein biosynthesis [2]. Niche-resident senescent cells impair cellular functionality and population dynamics, reducing regenerative capacity. Progressive senescent cell accumulation alters the tissue microenvironment through dysregulated cytokine production and signaling pathway modulation, ultimately impairing tissue regeneration [3, 4].

This review explores the concept of the muscle regenerative niche, including its cellular composition, signaling networks, and the characteristics of cellular senescence within the niche. Furthermore, we focus on the impact of niche cell senescence on muscle regeneration and explore potential therapeutic strategies for enhancing muscle regeneration.

## 2. Muscle regenerative niche and niche cell components

**Figure 1.**
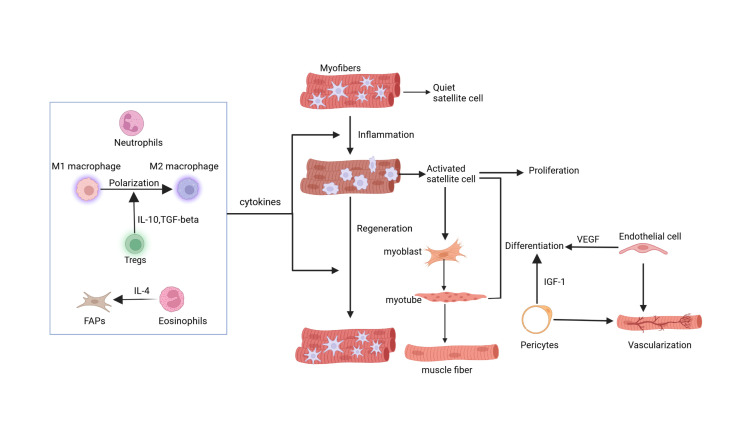
**Niche cells and muscle regeneration**. After muscle injury, inflammation occurs first, and satellite cells are activated from a quiescent state. Part of the activated satellite cells is responsible for proliferation to increase the number of cells. Other satellite cells differentiate into myoblasts, fuse to form muscle tubes, and ultimately develop into muscle fibers. Niche cells also play a role in regulating the muscle regeneration process. Macrophages, FAPs, Tregs, and other cells release cytokines that influence both the inflammatory and regenerative phases of muscle regeneration. In addition, niche cells can communicate with each other to more finely regulate muscle regeneration. Tregs promote macrophage polarization by secreting IL-10 and TGF-β, and eosinophils enhance FAPs proliferation through IL-4. Furthermore, endothelial cells and pericytes affect satellite cell differentiation and the vascularization of skeletal muscle.

The muscle regenerative niche constitutes a specialized microenvironment essential for tissue repair and regeneration. This niche comprises cellular components, extracellular matrix (ECM), vascular networks, and signaling molecules. Distinct cell populations within the niche execute specialized functions [1, 4]. Inflammatory cells clear damaged tissue and modulate the inflammatory response during muscle regeneration initiation, guiding the repair process. Satellite cells are activated, begin to proliferate and differentiate rapidly, driving muscle tissue repair and regeneration. The niche provides structural scaffolding and cellular guidance. Vascular networks transport vital nutrients and oxygen, promoting muscle tissue growth and repair, while also clearing metabolic waste and debris. Cell factors coordinate cellular interactions and behaviors, ensuring effective muscle repair and regeneration. Through their synergistic efforts, these components of the muscle regenerative niche create optimal conditions for muscle regeneration, aiding in the efficient repair and restoration of damaged muscle tissue [1, 4] (Fig. 1).

The muscle regeneration niche encompasses diverse cell populations that maintain tissue homeostasis and mediate adaptive responses. Satellite cells, the niche's essential components, maintain quiescence between the basal lamina and sarcolemma [3, 4]. The niche composition extends to muscle fibers, immune cells (macrophages, T/B cells, neutrophils), vascular components (endothelial cells), and stromal cells (FAPs, Schwann cells) [4]. Single-cell RNA sequencing and mass cytometry analyses have delineated ten distinct mononuclear cell populations in adult mouse muscle. Besides the mentioned cells, these techniques has revealed the presence of tenocytes and smooth muscle interstitial cells (SMMCs) in the muscle interstitial compartment [5]. SMMCs enhance muscle regeneration in both *in vivo* and *in vitro* settings, but their myogenic potential is evident only *in vitro* and is less apparent *in vivo*. Tenocytes may participate in ECM remodeling during regeneration. However, the current study does not classify tendon cells and SMMCs as integral components of the muscle regeneration niche. Although experiments suggest these cells influence muscle regeneration, the precise mechanisms are yet to be determined, necessitating further investigation [3-5].

## 3. Niche cells are involved in muscle regeneration

Muscle regeneration is a tightly coordinated process consisting of three successive, interrelated stages, including damaged muscle necrosis and inflammation, myofiber regeneration, maturation of newly formed myofibers, and remodeling of regenerated muscle [1, 6]. Throughout these phases, niche-resident cells execute specialized functions through coordinated interactions to orchestrate regeneration.

As the key component of niche cells, recruitment of leukocytes is essential for necrosis and inflammation formation in injured muscles [1]. Neutrophils are the initial inflammatory cells to be recruited to the site of muscle injury, typically within 6 hours post-injury [7]. Subsequently, macrophage infiltration follows, peaking at 2-4 days post-injury [1]. Macrophages exhibit polarization into two functionally distinct subsets: pro-inflammatory and anti-inflammatory phenotypes. The macrophages that infiltrate the muscle early on are pro-inflammatory CD68^+^/CD163^-^ M1 macrophages, followed by anti-inflammatory CD68^-^/CD163^+^ M2 macrophages [7]. M1 macrophages (peaking at 24h post-injury) mediate tissue debridement through phagocytosis and pro-inflammatory cytokine production (TNFα, IL-1). These cells execute myofiber lysis and cellular debris clearance. During regeneration, M1 macrophages help to recruit satellite cells to the injury site and stimulate their proliferation [8, 9]. M2 macrophages secrete anti-inflammatory cytokines, such as IL-10, and promote the proliferation and differentiation of satellite cells [10]. Regulatory T cells (Tregs) critically regulate macrophage polarization dynamics within the niche [11]. Tregs modulate regeneration through immunosuppressive cytokine secretion and inflammatory microenvironment regulation. Treg-derived IL-10 and TGF-β drive anti-inflammatory macrophage polarization [12]. Fibro/adipogenic progenitors (FAPs) sustain Treg viability through IL-33/ST2 signaling and facilitate recruitment via calcitonin gene-related peptide (CGRP) paracrine mechanisms [12-14]. Furthermore, studies reveal that IL-4, secreted by infiltrating eosinophils, activates FAPs in a manner dependent on IL-4. The IL-4/IL-13 signaling pathway in FAPs enhances proliferation and inhibits their differentiation into adipocytes, thus promoting muscle regeneration [15]. These niche cells interact to form new muscle fibers.

The regeneration phase features satellite cell activation and differentiation, representing the niche's core functional unit. Quiescent satellite cells undergo cell cycle re-entry, upregulate MyoD expression, migrate to injury sites, and either fuse with damaged fibers or commit to myogenic progenitor fates. Satellite cell migration is regulated by myofiber-derived signals, particularly Ephrin and Wnt7a pathways [16, 17]. Furthermore, Pax7 and Pax3 transcription factors orchestrate myogenesis by activating proliferation/commitment genes while repressing differentiation programs. Myogenic regulatory factors (MRFs) promote myogenic differentiation, which are located downstream of Pax7 and Pax3 [7, 18]. In addition, during the inflammatory phase, prostaglandin E2 (PGE2), produced by muscle fibers and other niche cells, binds to EP4 receptors on satellite cells. This signaling cascade activates cAMP/phosphoCREB and induces Nurr1 expression, driving muscle stem cell expansion [19, 20]. Moreover, pericytes facilitate adult muscle differentiation through insulin-like growth factor 1 (IGF1) and maintain quiescence *via* angiopoietin 1 (ANGPT1) [21]. Additionally, satellite cells highly express VEGF (vascular endothelial growth factor), critical for recruiting vascular endothelial cells to support angiogenesis and adequate capillarization of skeletal muscle fibers [22]. During the final differentiation phase, myogenic progenitors form elongated myoblasts that fuse into multinucleated myotubes. These myotubes complete terminal differentiation, maturing into functional myofibers. Terminal differentiation is regulated by specific factors including KLF5 and Sema4C [23, 24]. Niche cell interactions represent the cornerstone of muscle regeneration. Deciphering their composition, functions, and regulatory networks is crucial for developing targeted therapeutic strategies.

## 4. Cellular senescence in muscle regeneration niche

### 4.1 The characteristics of senescent cells

**Figure 2.**
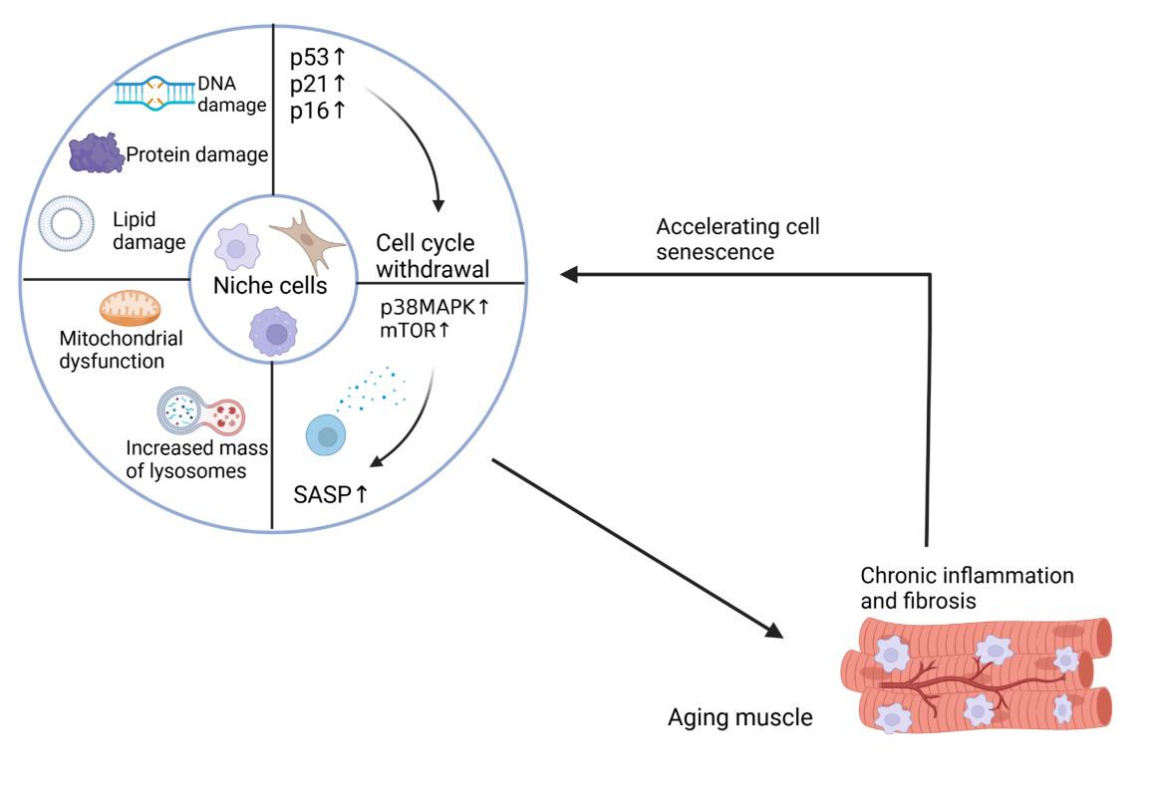
**The characteristics of senescent niche cells**. (1) Cell cycle withdrawal caused by up-regulation of p53, p21, p16, etc. (2) Damage to cellular molecules, including DNA, proteins, lipids. (3) Mitochondrial damage and increased lysosome content. (4) Upregulation of p38 and mTOR leads to increased secretion of SASP. Senescent niche cells affect skeletal muscle, creating a chronic inflammatory and fibrotic environment that accelerates the senescence of niche cells.

Currently, no universal biomarkers for senescent cells have been identified, but these cells exhibit some common features, such as cell cycle arrest, DNA damage response (DDR), β-galactosidase activity, and pro-inflammatory senescence-associated secretory phenotype (SASP) [2, 25] (Fig. 2). A common feature of senescent cells is irreversible cell cycle arrest, which may be an alarm response triggered by stress. This arrest state differs fundamentally from quiescence and terminal differentiation [26]. RB1 and its family members p107 (RBL1) and p130 (RBL2) are phosphorylated by specific cyclin-dependent kinases (CDKs; CDK4, CDK6, CDK2). This phosphorylation reduces the ability of RB family members to inhibit the activity of E2F family transcription factors, which are required for cell cycle progression [25, 27]. The CDK inhibitor p27KIP1, along with other RB family proteins such as p130 and p107, function as cell cycle regulators that maintain cell quiescence. In contrast, RB and p16INK4A, with their similar biochemical activities, are primarily linked to the maintenance of an irreversible cell cycle exit [27].

Senescent cells accumulate CDK inhibitors p21WAF1/Cip1 (CDKN1A) and p16INK4A (CDKN2A), which target CDK2 and CDK4/6 respectively. This process induces persistent RB activation, inhibits E2F transcriptional activity, and enforces cell cycle arrest [28]. The p53-dependent induction of p21CIP1 can drive cellular senescence from either the G1 or G2 phase of the cell cycle. Senescence maintenance depends on persistent p16INK4A expression, CDK4/6 inactivation, and hypophosphorylated RB activity, rather than p21CIP1 [27]. Notably, the ARF protein, an alternate reading frame protein derived from the p16INK4a gene locus that activates p53, plays a significant role in regulating cell cycle arrest in senescent mouse cells [25, 27]. Furthermore, characteristics of senescent cell cycle arrest encompass impaired ribosome biogenesis factors and the suppression of retrotransposons [29, 30].

The senescence-associated secretory phenotype (SASP) constitutes a hallmark of senescent cells and mediates many of their patho-physiological effects. Senescent cells secrete SASP components including chemokines, cytokines, proteases, growth factors, and other bioactive molecules [25]. SASP reinforces and propagates senescence in an autocrine and paracrine manner and stimulates immune responses to clear senescent cells. Furthermore, SASP factors mediate wound healing, and tissue plasticity and also contribute to persistent chronic inflammation [25].

SASP is regulated by enhancer remodeling and activation of transcription factors such as NF-κB, C/EBPβ, GATA4, mammalian target of rapamycin (mTOR) and p38MAPK signaling pathways [31-33]. In senescent cells, over-activation of mTOR boosts the rate of protein synthesis, contributing to the production of SASP. The over-activation of mTOR affects the proteins synthesized, resulting in high protein production and lower protein quality in senescent cells [34]. Over-activation of mTOR accelerates ribosome elongation but reduces translation fidelity, increasing the error rate and yielding more low-quality proteins [35]. Additionally, mTOR over-activation enhances ribosome biogenesis, potentially inducing cell cycle arrest [30, 34].

Cellular senescence triggers DNA damage, including oxidative modifications, single-strand breaks (SSBs), double-strand breaks (DSBs), and mutations [34, 36]. DSBs in nontelomeric regions of chromosomes results in damage through mutations and increased genomic instability. The repair of DSBs *via* DNA end-joining is less efficient, characterized by reduced fidelity, which contributes to a faster accumulation of sequence errors and further genomic instability [34]. Telomere shortening is another primary indicator of DNA damage. Telomeres, looped and repetitive DNA structures located at chromosome ends, are stabilized by the Shelterin protein complex. This organization prevents telomeres from being recognized by the DNA damage response (DDR) and double-stranded DNA break (DSB) repair mechanisms [25, 37]. During cell proliferation, telomeres shorten, causing telomeric DNA loop instability and telomere decapitation. This generates telomere dysfunction-inducing foci (TIFs), which trigger the DDR and result in cell cycle arrest. Beyond telomere shortening, DNA damage can induce somatic mutations, which are manifestations of genomic instability [25].

Beyond DNA damage, macromolecular damage (proteins, lipids) provides additional diagnostic indicator for cellular senescence. Protein damage, a hallmark of cellular aging, correlates with increased ROS, diminished activity of the ubiquitin-proteasome system (UPS), reduced autophagy function, and downregulation of molecular chaperones [34]. Oxidative protein damage is largely irreversible, and ubiquitin proteasome system (UPS) or autophagic degradation usually eliminates these proteins. Given the activity of the UPS and autophagy in senescent cells, they could serve as diagnostic indicators of the senescence state [25]. Similarly, PML bodies, acting as sensors for ROS and oxidative damage, can function as non-exclusive biomarkers of cellular senescence [38, 39]. Moreover, cellular senescence induces lipid damage. Lipids maintain membrane integrity, mediate energy metabolism, and regulate cellular signaling. Mitochondrial dysfunction in senescent cells results in ROS-induced lipid damage, lipid deposition, and lipofuscin accumulation [40, 41].

Senescent cells display significant mitochondrial alterations affecting function, dynamics, and morphology. Mitochondrial dysfunction in senescence is characterized by impaired respiratory capacity and decreased membrane potential. Mitochondrial dysfunction modulates diverse senescent phenotypes through multiple signaling pathways. Impaired OXPHOS generates excessive ROS, inducing DNA/protein/lipid damage, telomere attrition, and DDR activation [25, 42, 43]. Subsequently, the accumulation of damaged DNA with aging triggers PARP1 activation, which depletes the NAD pool in a PARP1-dependent manner and restricts the NAD availability for sirtuins, contributing to mitochondrial dysfunction [42]. Cells with a decreased NAD+/NADH ratio exhibit elevated ADP/ATP and AMP/ATP ratios, activating AMPK, which is a critical sensor for energy deprivation and promotes cell cycle exit [25, 42, 44]. Furthermore, mitochondrial dysfunction in aging is associated with the regulation of the SASP, and mitophagy in senescent cells seems to suppress the SASP [25].

Lysosomes play a crucial role in maintaining protein homeostasis, organelle quality control, and resolving cellular stress. Senescent cells exhibit lysosomal biogenesis and hypertrophy [25, 45]. However, this does not necessarily correlate with increased activity. Despite enhanced lysosomal biogenesis and accelerated lysosomal substrate delivery, senescent cells paradoxically show accumulation of lipofuscin and senescence-associated β-galactosidase, within lysosomes, along with neutralization of lysosomal PH [45]. This dysfunction likely reflects progressive macromolecular damage exceeding lysosomal clearance capacity. Consequently, cells accumulate progressively more cellular damage over time, characteristic of cellular senescence. Over time, the increased mass of lysosomes and lipofuscin accumulation may indicate a more advanced state of senescence, potentially compromising lysosomal integrity and activity, and exacerbating cellular aging [45, 46]. In addition, cellular senescence causes changes in chromatin. During senescence, cells undergo epigenetic modifications, an overall increase in chromatin accessibility, changes in chromatin morphology, and senescence-associated distension of satellites (SADSs) [25].

### 4.2 Characteristics of cellular senescence in muscle regeneration niche

The muscle regeneration niche exhibits cellular senescence characterized by morphological and functional alterations in satellite cells, FAPs, and other niche components, driven by aging and diverse senescence-inducing factors. Studies reveal that while aged muscle regenerative niche cells display heterogeneity, the genes differentially expressed across conditions typically share two traits: inflammation and fibrosis. Enrichment analysis of signaling pathways and transcription factors indicates that inflammatory responses and extracellular matrix remodeling/fibrotic reactions in senescent niche cells are significantly upregulated under different conditions. Core cellular processes in aged cells, including gene expression, splicing, translation, cell cycle progression, and DNA replication and repair, are downregulated across different conditions. In contrast to non-senescent cells, senescent cells show an enrichment in stress-related pathways, including metabolic stress responses like ROS production, oxidative phosphorylation, lipid transport, and metabolism, whereas DNA repair and mitochondrial function pathways are suppressed, and all exhibit increased DNA damage foci and telomere damage responses [47].

FACS-isolated SA-β-gal+ cells from injured muscle were subjected to scRNA-seq, generating an in vivo senescence atlas that identified three major senescent populations (satellite cells, macrophages, FAPs) and minor cell fractions. Nevertheless, classic aging markers like CDKN2A, p21CIP1, IL-6, and IL-1 cannot be observed in all *in vivo* senescent niche cells [47]. A possible explanation is that the aging status *in vivo* may be influenced by trigger factors, aging cells, aging environment, and the temporal resolution of the immune system, leading to the emergence of various phenotypic characteristics. Alternatively, SA-β-gal+ populations may contain non-senescent subsets with distinct transcriptional profiles. These experiments further demonstrate the common senescent characteristics of muscle regeneration niche cells. However, these experimental methods do not cover all cells in the niche but focus on the main cell populations (satellite cells, mononuclear/macrophages, FAPs). Despite this limitation, consistent senescence phenotypes in major populations suggest comparable traits may exist in other niche cells.

Besides the intrinsic changes in regenerative niche cells, the alterations in the niche environment due to senescence also constitute a significant feature. These environmental changes can affect the functionality of non-senescent cells, primarily by inducing senescence through the action of the SASP, which includes a multitude of chemokines, inflammatory cytokines, proteases, growth factors, and numerous additional molecules [48, 49]. SASP accelerates cell senescence, and cell senescence produces SASP, forming a vicious cycle [50]. Researchers used an improved FunRes algorithm to reconstruct the ligand-receptor interactions between senescent cells and non-senescent satellite cells. Subsequently, signal pathway impact analysis (SPIA) is applied to the transcription factors associated with these interactions. The results indicate that the SASP produced by senescent cells activates the downstream signaling pathways (senescence, apoptosis, and inflammation) of non-senescent SCs and inhibits the cell cycle and proliferation signaling pathways (MAPK and AKT signaling pathways). This paracrine mechanism induces proliferation arrest and secondary senescence in neighboring cells [47]. Collectively, senescent niche cells and their altered microenvironment synergistically compromise muscle regenerative potential.

### 3.3 Cell population changes in the senescent muscle regeneration niche

Beyond senescent cell accumulation, aging induces significant shifts in niche cell composition and subpopulation dynamics. These changes help elucidate the detrimental impact of aging on skeletal muscle regeneration. Single-cell sequencing shows that in aging skeletal muscle, the number of B cells and T cells significantly increases, while endothelial cells and Schwann cells are depleted. NK cell numbers increased in aging human skeletal muscle but not in mice [51, 52]. These changes in both species indicate that aging alters the muscle regeneration niche's cell population, providing initial insights into the causes of chronic inflammation and reduced vascularization in aging skeletal muscle. DEGs and KEGG analysis reveal activation of gene expression programs linked to age-related inflammation, including increased pro-inflammatory IL-6, further highlighting the heightened inflammation during muscle aging and more fully illustrating this phenomenon [51]. Age-related niche cell subtype alterations significantly impair regeneration, as detailed subsequently.

## 5. Senescent niche cells affect skeletal muscle regeneration

**Figure 3.**
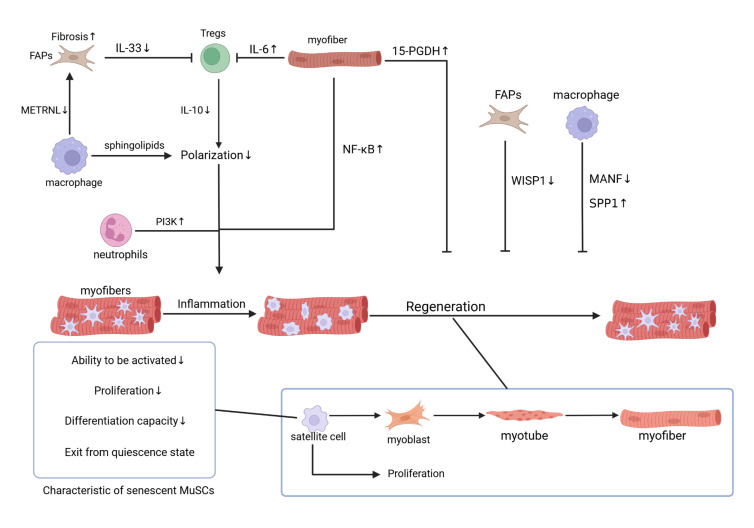
**Effects of senescent niche cells on muscle regeneration**. The impact of senescent satellite cells on muscle regeneration is characterized by a diminished capacity for activation, proliferation, differentiation, and maintenance of a quiescent state in senescent satellite cells. In addition, altered communication among senescent niche cells influences muscle regeneration. Changes in cytokine secretion, reduced macrophage polarization to an anti-inflammatory phenotype, and increased fibrosis collectively prolong the inflammatory response and hinder muscle regeneration. Furthermore, senescent muscle fibers, FAPs, and macrophages exhibit changes such as alterations in 15-PGDH and WISP1, impacting muscle regeneration.

As previously stated, Skeletal muscle regeneration primarily involves satellite cell proliferation and differentiation. Evidence indicates that the conversion of satellite cell and other niche cells into senescent cells not only diminishes the regenerative progenitor pool but also conferring a senescence-like inflammatory niche to the tissue microenvironment, impacting muscle regeneration *via* pro-inflammatory SASP [47]. It is valuable to understand how senescent niche cells affect skeletal muscle regenerative repair. We detail the impact of senescent satellite cells on muscle regeneration and explore the role of other senescent niche cells in relation to satellite cells, illustrating their impact throughout the regenerative process (Fig. 3).

### Satellite Cell

While skeletal muscle exhibits robust regenerative capacity in healthy adults, satellite cell dysfunction in degenerative conditions and aging compromises tissue repair [53-57]. This impaired capacity manifests in several ways.

Decreased ability of senescent cells to be activated. Study by Leiter et al [58] indicates that satellite cells in young mice activate promptly upon stimulation, but this activation may be delayed for up to 24 hours in aged mice. This decline in activation may be related to the loss of the splicing factor SRSF1, which impairs satellite cells' ability to self-renew and differentiate after activation. SRSF1 is expressed in activated SCs and forms multipolar spindles by mediating fgfr1op2 splicing. SRSF1 is notably absent in quiescent SCs compared to activated satellite cells, indicating that it may be crucial for satellite cell activation [59].

Aging leads to a decrease in satellite cells [60], potentially due to impaired activation and proliferation in response to injury. Research has found that in elderly mice, quiescent satellite cells transition into an irreversible pre-senescence state due to the weakened inhibitory effect of p16INK4a, leading to the loss of reversible quiescence. Injury triggers accelerated senescence progression in these cells, persisting even in youthful microenvironments [54]. As aging progresses, the number of satellite cells (SCs) expressing the transcription factor Pax7 at high levels (Pax7Hi) significantly decreases. In older mice, exercise stimulates the expression of granulocyte colony-stimulating factor (G-CSF), which helps regulate age-related loss of Pax7Hi cells [61, 62]. Similarly, single-cell data show that aging causes a change in the proportion of satellite cells and a decline in the number of PAX7High MYF5Low cell subsets, which have a strong ability to asymmetrically divide to maintain a stable pool of satellite cells [63]. In addition, senescent satellite cells exhibit decreased NIMA-related kinase 2 (Nek2) levels, leading to spindle formation collapse, impaired asymmetric division, and increased apoptosis, which further deplete the satellite cell pool [64].

Age-related dysregulation of myogenic regulatory factors (MRFs) impairs satellite cell differentiation capacity [4]. Meanwhile, due to the limited proliferation of muscle stem cells (MuSCs) and myogenic progenitor cells, the differentiation of progenitor cells into new muscle fibers is delayed, leading to an increased degree of fibrosis [65]. Senescent MuSCs upregulate cellular communication network factor 2 (CCN2), a key mediator of myogenic differentiation impairment. CCN2 overexpression downregulates MHC expression and blocks terminal myogenic differentiation. In addition, CCN2 induces muscle stem cells to change from a myogenic state to a fibrotic state, and causes excessive proliferation and differentiation of fibroblast, leading to skeletal muscle fibrosis [66].

Aging leads to the exit of satellite cells from the quiescent state, which ultimately depletes the satellite cell pool and leads to a decrease in the number of satellite cells. The SPRY1 gene is a known quiescent regulator of muscle stem cells. Aging leads to an increase in methylation of the SPRY1 gene, which inhibits the replenishment of the stem cell pool, contributing to a diminished regenerative response [56, 67]. In addition, the withdrawal of the quiescent state may also be related to the change in the proportion of satellite cells, namely, the decrease of PAX7High MYF5Low cells in the deeper quiescent state and the increase of MYF5High cells that are more inclined to activate and commit to differentiation [63].

### Muscle Fibres

NF-κB, a transcription factor activated by cellular damage and stress, has increased activity with aging and aging-related chronic diseases. This activation is particularly pronounced in senescent myofibers [47, 68]. Paracrine NF-κB signaling from differentiated myofibers compromises muscle stem cell functionality through non-autonomous mechanisms. Typical NF-κB signaling is activated in proliferating myoblasts and inhibit myoblast differentiation, partially by suppressing expression of the myogenic transcription factor MyoD [69]. Tonic activation of typical NF-κB signaling in myofibers leads to progressive muscle atrophy, partly because of the increased levels of the E3 ubiquitin ligases MURF [70]. Conversely, inhibition of NF-κB activity in multiple cell types, including macrophages and myofibers, reduces inflammation and fibrosis and accelerates repair after muscle injury [71]. Moreover, Chronic inflammatory signaling in myofibers is mediated by NF-κB, which delays the myogenic potential of satellite cells in aging muscle [72].

FGF2 in myofibers activates muscle stem cells (MuSCs), while neutralizing FGF2 or blocking its receptors restores quiescence. In muscle stem cells of older adults, the FGF2 negative regulator sprouty1 exhibits increased DNA methylation, which affects the cells' transition to a quiescent state [56, 67]. Additionally, Prostaglandin E2 (PGE2) stimulates young MuSCs to mediate muscle repair [20]. Aging elevates 15-hydroxyprostaglandin dehydrogenase (15-PGDH), degrading PGE2 and impairing regeneration [19].

Senescent muscle fibers affect Tregs in the regeneration niche. Research has found that IL-6 from muscle fibers promotes the maturation of Tregs through the Areg/ST2/EGFR signaling pathway [73]. However, persistent IL-6 signaling due to chronic inflammation in aging muscle can impede Tregs differentiation, leading to elevated expression of IL-6 in aging muscle fibers and subsequent suppression of Treg function [74]. Consequently, senescent myofibers may disrupt macrophage polarization dynamics.

### Neutrophils

Senescence impairs neutrophil chemotaxis, exacerbating tissue damage and systemic inflammation during migration. The decline in chemotaxis correlates with the Phosphoinositide 3-kinase(PI3K) pathway, and targeted inhibition of PI3Kγ and PI3Kδ can effectively restore neutrophil migration [75]. Senescence also affects the apoptotic capacity of neutrophils. Macrophages preferentially remove apoptotic neutrophils, serving to abort the inflammatory state. After migration into the inflammatory environment, neutrophils are exposed to apoptosis-promoting and apoptosis-delaying signals. Senescence reduces the responsiveness of neutrophils to anti-apoptotic signals but has little effect on pro-apoptotic signals, resulting in disturbed neutrophil apoptosis, which affects the onset and regulation of the inflammatory response [76].

### Macrophages

Senescence impairs macrophage phagocytosis and polarization, resulting in sustained chronic low-grade inflammation. The phagocytosis of apoptotic cells by macrophages diminishes with age. This defect delays inflammation resolution and exacerbates tissue damage in aged organisms [76]. Single-cell RNA sequencing and flow cytometry reveal age-related macrophage polarization shifts: reduced LYVE1+ M2 reparative subsets and expanded LYVE1- M1 proinflammatory populations in murine skeletal muscle. The expression of M2 markers in skeletal muscle macrophages of aged mice is reduced, whereas that of pro-inflammatory and aging-related markers is elevated [77]. Furthermore, the pro-inflammatory cytokine SPP1 (osteopontin) is highly expressed in skeletal muscle macrophages of aged mice [77, 78]. After injury, SPP1 protein in the niche is significantly elevated and inhibits the muscle generating ability of SCs [78]. In addition, sphingolipids accumulates in aging muscle, inhibiting the polarization of macrophage M1 to M2, and further forming a pro-inflammatory environment [79].

The decline in satellite cell counts within aging muscles might stem from reduced macrophage-derived factors that promote cell proliferation. Muscle injury generates interferon-γ, stimulating interferon-responsive macrophages to produce the cytokine CXCL10, which promotes the proliferation of muscle satellite cells through CXCR3. Aged mice exhibit impaired regeneration due to downregulated interferon-γ response genes, compromising macrophage-mediated satellite cell activation [80]. Furthermore, another study indicates that the immune modulator mesencephalic astrocyte-derived neurotrophic factor (MANF) is induced following muscle damage in young mice, but not in aged animals, and its expression is crucial for successful regeneration. This mechanism is disrupted in aged animals and is dependent on the regulation of macrophage function and can be enhanced by the delivery of MANF. These studies further substantiate the close association between macrophages and satellite cells in aged muscle regeneration [81].

Compared with the classification of M1, M2 macrophages and monocytes, single-cell technology enables more detailed cell classification, allowing for a deeper understanding of aging-induced subgroup changes. Neuza S Sousa et al. categorized macrophages into five groups based on characteristic genes: infiltrating monocyte-derived macrophages (infMacs), regeneration-associated regulatory macrophages (regMacs), late regeneration-associated macrophages(lateMacs), repair-associated macrophages(repMacs), and intermediate macrophages (intMacs). Aged muscle shows reduced regMacs populations that normally express high levels of repair-promoting growth factors (Igf1, Vegfa, Pdgfa). Additionally, during aged muscle regeneration, a distinct 5dpi-specific subset emerges. This population, termed age-related macrophages (agedMacs), upregulates the expression of several chemokines, including Cxcl10, Ccl8, and Ccl5, as well as pro-fibrosis genes, potentially leading to prolonged inflammation and increased fibrosis. Single-cell sequencing-based macrophage classification greatly aids in studying the impact of aging on muscle regeneration [52]. Furthermore, macrophage subset characterization informs targeted senescent cell clearance strategies to enhance regeneration. This includes more targeted elimination of senescent cells, such as agedMacs, while preserving regMacs.

### Treg cells (Tregs)

Although Foxp3^+^CD4^+^ regulatory T cells (Tregs) are known for their ability to suppress immune responses, Dalia Burzyn et al. find that Tregs present in muscle are a specialized population. Their study indicates that a shift from a pro-inflammatory to a pro-regenerative phenotype in acutely injured skeletal muscle is accompanied by an increase in Tregs. Tregs promote muscle stem cell activation via macrophage polarization from M1 to M2 phenotypes [11]. Current research indicates that muscle Tregs regulate inflammation/fibrosis through IL-10 secretion and M2 macrophage polarization [12, 82]. Age-related Treg dysfunction prolongs inflammation, disrupts immune coordination, and compromises muscle regeneration [83]. It is speculated that the impact of senescent Tregs on macrophage polarization may be attributed to prolonged inflammation due to decreased IL-10 secretion [11]. Successful muscle regeneration has been shown to require timely infiltration and transition of M1 and M2-polarized macrophage phenotypes, and too many M1 macrophages and prematurely transformed M2 macrophages delay healing of muscle tissue. Therefore, attention to the regulation of senescent Tregs on phenotypic expression of macrophages is critical [10, 84]. In addition, aged skeletal muscle exhibits T cell accumulation that amplifies pro-inflammatory signaling via IL-2/STAT5 activation [52]. While Treg functions in muscle niches are well-characterized, the reciprocal impact of senescent Tregs on niche components remains underexplored. Exploring the influence of senescent Tregs on interactions with other muscle niche cells is a promising and valuable research avenue.

### Endothelial cells and pericytes

Satellite cells, located in close proximity to capillaries, engage in bidirectional signaling with endothelial cells. MuSCs recruit endothelial cells to support angiogenesis by expressing high levels of VEGFA. Endothelial cells induce quiescence in MuSCs by expressing DII4 via Dll4-Notch signaling and increased reserve cell formation in MuSCs after injury [22]. During muscle regeneration, endothelial cells secrete paracrine factors (Ang1, VEGF) that promote MuSCs proliferation and differentiation [22, 85]. Pericytes are mural cells surrounding blood vessels that are adjacent to endothelial cells. Pericytes play a key role in the maturation and maintenance of vascular branching morphogenesis. Pericytes facilitate myogenic differentiation *via* IGF-1 and induce quiescence *via* ANGPT1. Ablation of pericytes in mice leads to impaired muscle fiber atrophy and establishment of stem cell quiescence [21].

Study on the effects of senescent endothelial cells and pericytes on muscle regeneration niches is relatively limited. Current evidence suggests that the proliferation and differentiation of senescent pericytes are reduced, affecting muscle regeneration, and senescent endothelial cells may be related to the loss of dystrophin expression [65, 86]. Pericytes, which stabilize capillary structure by associating with capillary endothelial cells, play a crucial role in maintaining the vascularity of skeletal muscle. However, as skeletal muscle ages, its capillaries undergo a thinning process [65]. Therefore, we can speculate that the aging of pericytes and endothelial cells leads to changes in their communication with other niche cells. These alterations could affect blood vessel formation and the functionality of other niche cells within muscle regeneration niches. However, these hypotheses require validation through further experimentation.

### Fibro/Adipogenic Progenitors (FAPs)

FAPs are mesenchymal stromal cells (MSCs) characterized by high expression of PDGFR-α, which play an important role in homeostasis and repair in a wide range of tissues [87]. Conversely, in diseased or atrophic muscle, FAPs may induce fibrosis and the expense of adipogenesis, thereby impeding normal muscle regeneration [88, 89].

Undifferentiated FAPs enhance myogenic progenitor activation. Undifferentiated FAPs can induce differentiation of activated myoblasts by secreting molecules such as IL-6, IGF-1, Wnt1, Wnt3a and Wnt5a [90]. In damaged skeletal muscle, FAPs are recognized as the main source of fibroblasts [91]. After injury, FAPs become activated and proliferate, increasing their numbers in the necrotic region, which need to be removed promptly. Otherwise, they differentiate into fibroblasts and adipocytes [92]. Fibroblasts secrete extracellular matrix proteins and growth factors, differentiating into myofibroblasts that elevate α-smooth muscle actin (α-SMA) expression and ECM synthesis, ultimately leading to fibrosis [93]. Lack of TGF-β1 in macrophages inhibits FAP proliferation and reduces fibrosis [94]. Additionally, IL-4 can be released by infiltrating eosinophils, activates FAPs. IL-4/IL-13 signaling in FAPs contributes to proliferation and the inhibition of the lipogenic differentiation of FAPs, which in turn support myogenesis [15]. IL-1α and IL-1β inhibit the lipogenic differentiation of FAPs, whereas epidermal growth factor (EGF) and betacellulin (BTC) stimulate the proliferation of FAPs [95]. Inactivation of retinoic acid (RA) signaling in FAPs induces adipogenic differentiation, consequently increasing the proliferation of FAPs [92].

Study on the mouse model of muscular dystrophy indicates that senescent FAPs impact the functionality of muscle progenitor cells (MPCs) [96]. It has been shown that WNT1-induced signaling pathway protein 1 (WISP1) is a FAP-derived matricellular signaling that is lost during aging. The absence of WISP1 in FAPs leads to dysfunction of MuSCs in senescent skeletal muscle, which control the expansion and asymmetric commitment of muscle stem cells through Akt signaling [88]. Senescent FAPs also influence Tregs. Senescent FAPs modulate Treg dynamics: FAP-derived IL-33 recruits Tregs to injured muscle, while age-related FAP depletion impairs Treg recruitment. By supplementing IL-33 locally or systemically to regulate the function of immune cells, the regenerative ability of aging mice can be restored through the reconstruction of Treg recruitment after injury [13]. Furthermore, a recent study finds that METRNL protein (a muscle factor) is deficient in aging muscle after injury, and macrophages are the main source of METRNL. METRNL boosts immune responsiveness to counter the pro-fibrotic program by promoting TNF-induced apoptosis in FAPs. Treatment with recombinant METRNL has been shown to enhance muscle regeneration in aged mice [97]. These findings establish FAPs as therapeutic targets for aged muscle regeneration.

## 5. Signaling pathways of niche cellular senescence intervening in muscle regeneration

Signaling pathways are important to muscle regeneration, making it essential to explore how senescent cell signaling affects cells and muscle regeneration. As stated earlier, DNA damage activates the DDR, prompting H2AX phosphorylation that disrupts cell cycle checkpoint inhibitors like p16 and p53 [98]. This activates p53/p21 and p16INK4a/pRB pathways, forming an integrated network that arrests cell cycle progression [25]. Furthermore, the Wnt and Notch signaling pathways regulate satellite cell proliferation and differentiation, playing crucial roles in muscle regeneration. The subsequent sections detail the alterations in these signaling pathways during cellular senescence and their impact on muscle regeneration.

### p53-p21 Pathway

P53-dependent senescence in niche cells suppresses muscle regeneration through multiple factors, including ROS. Studies have shown that the inactivation of Piezo1 increases the expression of T-type Ca2^+^ channels, enhances Ca2^+^ influx, leading to the accumulation of ROS and P53. This triggers p53-dependent senescence in satellite cells, compromising quiescence maintenance and impairing proliferative/differentiation capacity [99]. Within the regeneration niche, P53 directly regulates the MyoD-dependent transcription pathway, suppresses the expression of Myogenin, and modulates myoblast differentiation [7, 100]. Additionally, Numb deficiency elevates p53-dependent senescent cells in the niche, which recruit macrophages to sustain inflammatory states and promote fibrosis. [101].

### p16-pRB Pathway

Cellular senescence occurs through telomere shortening/p53-DDR or p16-dependent pathways [2]. p16, a crucial component of the pRB signaling pathway, inhibits cyclin-dependent kinases (CDKs), serving as a primary driver of cellular senescence [25]. p16INK4a prevents cell cycle progression by inhibiting CDK4/CDK6 expression, thereby preventing pRB activation; unphosphorylated RB then binds to E2F, inhibiting its activation [102]. Additionally, activation of the INK4/ARF locus is a critical sensor of senescence within the p16INK4a/pRB pathway. The INK4/ARF locus connects the p16INK4a/pRB pathway with the p53/p21CIP1 pathway. ARF represses MDM2, which prevents the expression of p53, creating an important link between the p16INK4a/pRB pathway and the p53/p21CIP1 pathway [2].

Niche cells undergo p16-mediated senescence, confirmed by p16INK4a upregulation in immunocytochemical analyses. Senescent niche cells that are p16-dependent influence muscle regeneration [47]. Increased p16 expression induces senescence in quiescent satellite cells, compromising activation and regenerative potential [54]. Upregulation of the p16INK4a pathway can not only directly induce muscle stem cell senescence but also indirectly induce muscle stem cell senescence by reducing mitochondrial membrane potential and activating the mitochondrial pathway [103]. Moreover, p16INK4a inhibition rejuvenates atrophied muscle. Sousa-Victor et al. delivered short hairpin RNA (shRNA) targeting p16INK4a (p16INK4a shRNA) into stationary senile satellite cells, causing the satellite cells to downregulate p16INK4a expression and significantly restore early activation after injury, while reducing the expression of age-related genes [54]. These experiments demonstrate the significant role of the p16 signaling pathway in muscle regeneration, highlighting the value of strategies promoting muscle regeneration targeting p16.

### P38 MAPK Pathway

The DNA damage response (DDR) stimulates SASP secretion, primarily through p53-dependent pathways. However, Freund et al. indicate that the p38MAPK/NF-κB pathway is a key regulatory pathway for SASP. Experimental data indicate that the kinetics of p38MAPK activation is closely related to the developmental kinetics of SASP [31]. p38MAPK induces SASP through transcriptional upregulation of SASP components. p38MAPK-mediated SASP regulation operates independently of DDR, while DDR-activated p53 signaling inhibits p38MAPK [31].

Cellular senescence within the muscle niche activates p38 MAPK expression, impacting muscle regeneration. Bernet et al. collect and quantitatively analyze RNA from FACS-isolated satellite cells, discovering that senescent SCs exhibit p38α/β MAPK activation. p38α/β hyperactivation impairs self-renewal, while p38 inhibition restores asymmetric division and regenerative capacity by downregulating cell cycle inhibitors [104, 105]. Furthermore, recent study has indicated that aging increases circulating levels of endothelin-1 (ET-1), which inhibits myoblast differentiation through ETB receptor and p38 MAPK signaling [106]. These findings establish p38 signaling as a promising target for enhancing muscle regeneration.

### Notch Pathway

Notch signaling is a major pathway that regulates satellite cell self-renewal and differentiation to maintain the quiescent state and homeostasis [107]. Disruption of canonical Notch signaling triggers spontaneous satellite cell differentiation and stem cell pool depletion [107-109]. Notch signaling is regulated by multiple factors. For example, Notch help maintain the quiescent state of SCs through the Notch-collagen V-Calcitonin receptor signaling cascade [108]. Additionally, the recombining binding protein-Jκ (RBP-Jκ) is a nuclear factor required to mediate Notch signaling. Its deletion in satellite cells results in spontaneous differentiation and a progressive loss of MuSC pool [107]. Furthermore, the transcription factor FoxO3 is expressed in quiescent MuSCs and supports quiescence by activating Notch signaling [110]. Senescent satellite cells have intact Notch receptors, but the delta ligand levels decrease with age, leading to a diminished capacity for activation [111]. Irina M Conboy et al. find that exposure to a youthful systemic environment rejuvenated senescent satellite cells. Young serum exposure upregulates Delta ligand expression, enhances Notch activation, and restores proliferation in aged satellite cells. Furthermore, forced activation of Notch can restore the activation and regenerative potential of aged satellite cells, suggesting that the intrinsic regenerative capacity of aged satellite cells may still be intact [111, 112].

Endogenous pSmad3 and active Notch antagonize each other in controlling satellite cell proliferation, and the balance between them controls the regenerative capacity of muscle stem cells. Mechanistically, the Smad2/3 pathway plays an important role in TGF-β signaling and inhibition of myogenesis [113, 114]. Studies have shown that aging leads to increased levels of TGF-β and decreased activation of Notch signaling. Notch activation blocks TGF-β-dependent upregulation of CDK inhibitors p15, p16, p21 and p27, while Notch inhibition induces them. Therefore, the imbalance between pSmad3 and Notch in old SCs lead to the failure of satellite cell regeneration potential, while weakening TGF-β/pSmad3 in aging and injured muscles can restore the ability [113]. The transcription factor CCAAT/enhancer binding protein beta (C/EBP-β), expressed in satellite cells of healthy muscle, inhibits myogenic differentiation by suppressing MyoD. The inflammatory microRNA (miR)-155 promotes the differentiation process by inhibiting C/EBP-β, while its expression is suppressed by Notch1. In aging muscle, Notch1 signaling diminishes, leading to upregulation of miR-155 and downregulation of C/EBP-β, which contributes to satellite cell dysfunction [115, 116].

### Wnt Pathway

The balance between Notch and Wnt signals regulates the proliferation and differentiation of satellite cells. The initiation of differentiation is due to the transition of myoblast progenitors from Notch to Wnt signaling, correlating with elevated Wnt expression in tissues and heightened progenitor cell responsiveness to Wnt. Notch maintains GSK3β active while Wnt inhibits GSK3β in a typical cascade of Wnt signals. After Wnt activation, GSK3β's phosphorylation of β-catenin is suppressed, permitting β-catenin's nuclear translocation and influencing skeletal muscle repair [117, 118]. A decrease in GSK3β and an increase in β-catenin and Axin 2 expression, which are downstream targets of Wnt signaling, were observed in senescent satellite cells compared to their younger counterparts [119]. Furthermore, serum from aged mice contained components that activate Wnt signaling *via* Frizzled receptor, further disrupting the balance between Notch and Wnt signaling [119]. These factors contribute to the diminished myogenic potential in senescent satellite cells and induce a transform from myogenic to fibrogenic lineage [119]. Additionally, Wnt-3a upregulates CCN1 in aged muscle, elevating p53/p16/pRB levels and suppressing progenitor proliferation [120].

The Hox gene family, crucial for stem cell and tissue patterning during embryogenesis, can have its expression dysregulated in activated satellite cells of older mice, specifically inducing Hoxa9. Hoxa9 activates various developmental pathways such as Wnt, TGF-β and JAK/STAT that inhibit satellite cell function in aging muscle. Inhibition of Hoxa9 enhances satellite cell function and muscle regeneration in older mice, whereas its overexpression in young mice's satellite cells induces functional declines akin to those observed in aging [57]. Thus, Wnt signaling inhibition represents a promising strategy to mitigate age-impaired muscle regeneration.

## 7. Strategies for muscle regeneration by regulating niche cellular senescence

Skeletal muscle is the most abundant tissue in the human body, accounting for approximately 40% of body mass [1, 3, 4]. Muscle disorders are a growing concern, with numerous therapeutic strategies being explored to enhance muscle regeneration. Therefore, exploring methods to enhance muscle regeneration through the modulation of niche cell senescence represents a valuable and promising research avenue. The following section discusses therapeutic strategies targeting senescent niche cells to promote regeneration.

### 7.1 Senescent cell elimination

Removing senescent cells is an effective way to promote skeletal muscle regeneration, as these cells impede regeneration. By eliminating the negatively affected niche cells, regeneration is facilitated. In dystrophic models, senescent FAPs impair muscle progenitor cell function, and partially ablating FAPs with the anti-aging drug Fexetine can mitigate muscle damage and delay muscle function decline [96]. Dungan et al. show that eliminating SAβ-Gal^+^ cells with senolytic agents enhances muscle regeneration in aged mice [48]. However, Irina M. Conboy et al. discover that a youthful systemic environment can rejuvenate aging satellite cells [112]. This study indicates that the detrimental effects of senescent cells on the muscle regeneration niche are also linked to the local microenvironment. If the senescent cells are only eliminated to improve the muscle regeneration ability, the potential role of senescent niche cells in the muscle regeneration may be overlooked.

### 7.2 Cytokines

Modulating cytokines to regulate cell function is a therapeutic strategy. For example, the accumulation of sphingolipids in aged muscle can be mitigated by myriocin, which inhibits serine palmitoyltransferase (SPT) and thus promotes the polarization from pro-inflammatory M1 to anti-inflammatory M2 macrophages, consequently reducing inflammation [79, 121]. Additionally, since a large number of genes involved in lipid metabolism and lipid transport are up-regulated in senescent cells, researchers have found that CD36 may regulate the senescent secretory program *in vivo* and affect muscle regeneration. Blockade of CD36 improved muscle regeneration in both young and old mice, while also reducing inflammation and fibrosis [47]. Furthermore, IL-33 supplementation restores aged muscle regeneration by reconstituting Treg recruitment post-injury [13].

Cytokine regulation, which alters cell communication and mitigates the detrimental effects of senescent cells on other niche cells and niches, is a viable therapeutic approach for muscle regeneration. Compared to eliminating senescent cells, this approach considers the muscle regeneration effects of senescent cells after regulation. However, identifying key factors is challenging due to the complex and systemic nature of cytokines altered by senescent cells in muscle regeneration niches. Comparative therapeutic efficacy versus senescent cell clearance requires further investigation.

### 7.3 The modulation of the special signaling pathways

Aging leads to the upregulation of related signaling pathways, such as p38 and p53 signaling, inducing cell cycle arrest, SASP secretion, and chronic inflammation. Targeting these pathways represents a promising anti-aging strategy for enhancing muscle regeneration. Bernet et al. collect RNA from FACS-isolated satellite cells and find that senescent SCs exhibit abnormal p38α/β MAPK activation. p38 MAPK inhibition rescues senescent cells by downregulating cell-cycle inhibitors, such as p16INK4a, and enhancing their asymmetric division and regenerative capacity [104, 105]. Their work support that the kinetics of p38MAPK activation is closely related to the kinetics of SASP development, and SB203580-mediated inhibition reduces SASP secretion [105]. p38MAPK primarily induces SASP through NF-κB signaling. Inhibition of NF-κB or its downstream target phospholipase A2 (PLA2G5) in muscle fibers rescues the muscle regeneration potential of aging muscles. Similarly, NF-κB inhibition in various cell types, such as macrophages and myofibers, mitigates inflammation and fibrosis, and hastens muscle repair post-injury [31, 72]. Moreover, p53 inhibition can mitigate MuSCs senescence. Pharmacological suppression of P53 can decrease the formation of senescent MuSCs and enhance the muscle regeneration capacity of mice [99, 101]. These findings establish signaling pathway modulation as an effective therapeutic strategy.

### 7.4 Cell reprogramming

Cellular reprogramming offers distinct therapeutic advantages over senescent niche cell modulation for muscle regeneration. A study by Ori Bar-Nur et al. implies that MyoD combined with exposure to small molecules reprograms fibroblasts into expandable induced myogenic progenitor cells (iMPCs). These iMPCs exhibit self-renewal capacity and express satellite cell/myofibroblast markers [122]. Furthermore, Myofiber-specific expression of the Yamanaka reprogramming factor (Oct-3/4, Sox2, Klf4 and c-Myc [OSKM]) reduces the secretion of Wnt4, which promotes muscle stem cell quiescence, and thus can activate MyoD and Yap to cause activation of SCs and accelerate muscle regeneration [123].

### 7.5 Intervening metabolism

Skeletal muscle regeneration is metabolically regulated, making metabolic intervention a critical therapeutic strategy. Aging impairs autophagy, disrupting apoptotic homeostasis. AMPK-mediated p27Kip1 phosphorylation at Thr198 activates autophagy and suppresses apoptosis. Studies have shown that activating this pathway enhances the survival and proliferation of aged MuSCs, reduces cellular senescence, and improves transplantation outcomes [124]. Additionally, cytoplasmic STAT3 induces autophagy by dissociating protein kinase R (PKR) and preventing the phosphorylation of eukaryotic translation initiation factor 2α (eIF2α) [125]. STAT3 inhibition can activate autophagy at the nuclear level by promoting the transcription of autophagy-associated genes, and at the cytoplasmic level by STAT3/PKR phosphorylation targeting eIF2α, thereby enhancing MuSC muscle regeneration capacity [126].

Mitochondrial dysfunction impairs energy metabolism and exacerbates oxidative damage. Carbon monoxide (CO) increases PGC-1α levels in C2C12 myoblasts, thereby enhancing mitochondrial biogenesis and energy production. In addition, CO reduces oxidative stress damage by activating Akt signaling and inhibiting the muscle atrophy factor myostatin and atrogin-1. Through CO-loaded red blood cells (CO-RBCs), CO can be effectively supplemented to promote muscle regeneration [127].

The serine biosynthesis pathway (SBP) is a branch of glycolysis, and Psat1 is one of three SBP enzymes that control MuSC activation and myogenic progenitor cell expansion by producing the metabolite α-ketoglutarate (α-KG) and α-KG-generated glutamine. Exogenous supplementation of α-KG or glutamine improves age-related declines in muscle regeneration [128]. This indicates that interventions targeting metabolic pathways can alleviate the negative effects of aging and restore muscle regeneration.

## Conclusions

This review outlines the concept, composition, and function of the muscle regeneration niche and emphasizes the role of niche cells in muscle repair. It further examines how cellular senescence impacts niche microenvironment and cellular functionality. The review also discusses senescent cell-mediated signaling pathways in muscle regeneration and proposes regenerative strategies. While this review advances our understanding of niche and aging impacts on regeneration, key mechanisms remain elusive, including specific cell type roles and senescent cell effects. Future studies should elucidate these complex networks and develop advanced tools for age-related muscle regeneration.

## Data Availability

All data generated or analyzed during this study are included in this published article.
